# Draft Genome Sequence of the Oleaginous Yeast *Cryptococcus curvatus* ATCC 20509

**DOI:** 10.1128/genomeA.01235-16

**Published:** 2016-11-03

**Authors:** Dan Close, John Ojumu

**Affiliations:** Biosciences Division, Oak Ridge National Laboratory, Oak Ridge, Tennessee, USA

## Abstract

*Cryptococcus curvatus* ATCC 20509 is a commonly used nonmodel oleaginous yeast capable of converting a variety of carbon sources into fatty acids. Here, we present the draft genome sequence of this popular organism to provide a means for more in-depth studies of its fatty acid production potential.

## GENOME ANNOUNCEMENT

*Cryptococcus curvatus* ATCC 20509 is an oleaginous yeast strain capable of assimilating xylose, lactose, glucose, and sucrose, as well as a variety of agricultural and food-processing wastes, as carbon sources (1–3). Lipid production in *C. curvatus* ATCC 20509 can be induced under nitrogen-limiting conditions (4) and favors the synthesis of 18-carbon-chain-length fatty acids (5). The utility of these fatty acids for the production of biofuels and other high-value products, in tandem with its ability to grow using low- or negative-cost feedstocks, makes it a potential candidate for use in industrial fermentation processes. In this context, the *C. curvatus* ATCC 20509 draft genome sequence provides a supplemental point of comparison in relation to other oleaginous yeasts for elucidating the genetic mechanisms underlying fatty acid synthesis potentials and the metabolic controls governing the enactment of oleaginous metabolism.

To prepare for sequencing, *C. curvatus* ATCC 20509 was grown under standard laboratory conditions in YPD broth (10 g/liter yeast extract, 20 g/liter peptone, 20 g/liter glucose) at 25°C and 150 rpm. Cells were lysed during mid-log-phase growth by treatment with a buffer containing 8 M urea, 0.5 M NaCl, 20 mM Tris, 20 mM EDTA, and 2% SDS, and the DNA was isolated by phenol-chloroform–isoamyl alcohol extraction as previously described (6). Following isolation, RNA was removed via RNase treatment, and the DNA was ethanol precipitated. The purified DNA was used to generate shotgun and mate-pair libraries with insert sizes of approximately 323 bp and 6 kb, respectively. Genome sequencing was performed using an Illumina HiSeq 2500 platform. The shotgun library produced 6,150,342 reads. The mate-pair library produced 10,284,626 reads. The AllPaths LG whole-genome shotgun assembler (release version R49403) (7) was used for creation of a *de novo* genome assembly. This assembly represents an 87.8× coverage level from a total of 16 scaffolds (145 contigs). The average scaffold read length was 1.24 Mbp. The scaffold *N*_50_ was 3 Mbp, and the maximum scaffold length was 5.25 Mb. The total sequence length of the resulting draft genome was 19.9 Mbp, and the overall G+C content was determined to be 60.7%.

### Accession number(s).

This whole-genome shotgun project has been deposited in DDBJ/ENA/GenBank under accession no. MATS00000000. The version described in this paper is the first version, MATS01000000.
